# Blood Trimethylamine-N-Oxide Originates from Microbiota Mediated Breakdown of Phosphatidylcholine and Absorption from Small Intestine

**DOI:** 10.1371/journal.pone.0170742

**Published:** 2017-01-27

**Authors:** Wolfgang Stremmel, Kathrin V. Schmidt, Vera Schuhmann, Frank Kratzer, Sven F. Garbade, Claus-Dieter Langhans, Gert Fricker, Jürgen G. Okun

**Affiliations:** 1 Department of Gastroenterology, University Clinics of Heidelberg, Heidelberg, Germany; 2 Department of Pediatrics, University Clinics of Heidelberg, Heidelberg, Germany; 3 Institute of Pharmacy and Molecular Biotechnology, Ruprecht-Karls-University, Heidelberg, Germany; University of Hawai'i at Manoa College of Tropical Agriculture and Human Resources, UNITED STATES

## Abstract

Elevated serum trimethylamine-N-oxide (TMAO) was previously reported to be associated with an elevated risk for cardiovascular events. TMAO originates from the microbiota-dependent breakdown of food-derived phosphatidylcholine (PC) to trimethylamine (TMA), which is oxidized by hepatic flavin-containing monooxygenases to TMAO. Our aim was to investigate the predominant site of absorption of the bacterial PC-breakdown product TMA. A healthy human proband was exposed to 6.9 g native phosphatidylcholine, either without concomitant treatment or during application with the topical antibiotic rifaximin, or exposed only to 6.9 g of a delayed-release PC formulation. Plasma and urine concentrations of TMA and TMAO were determined by electrospray ionization tandem mass spectrometry (plasma) and gas chromatography-mass spectrometry (urine). Native PC administration without concomitant treatment resulted in peak plasma TMAO levels of 43 ± 8 μM at 12 h post-ingestion, which was reduced by concomitant rifaximin treatment to 22 ± 8 μM (p < 0.05). TMAO levels observed after delayed-release PC administration were 20 ± 3 μM (p < 0.001). Accordingly, the peak urinary concentration at 24 h post-exposure dropped from 252 ± 33 to 185 ± 31 mmol/mmol creatinine after rifaximin treatment. In contrast, delayed-release PC resulted in even more suppressed urinary TMAO levels after the initial 12-h observation period (143 ± 18 mmol/mmol creatinine) and thereafter remained within the control range (24 h: 97 ± 9 mmol/mmol creatinine, p < 0.001 24 h vs. 12 h), indicating a lack of substrate absorption in distal intestine and large bowel. Our results showed that the microbiota in the small intestine generated the PC breakdown product TMA. The resulting TMAO, as a cardiovascular risk factor, was suppressed by topical-acting antibiotics or when PC was presented in an intestinally delayed release preparation.

## Introduction

The small intestine is the main site of substrate absorption, whereas the colon absorbs mainly water and electrolytes (thereby promoting stool consolidation) and short-chain, microbiota-produced fatty acids as additional nutrients for mucosal epithelial cells. In addition to fibres and biliary dyes, the stool contains one trillion bacteria per gram for release from the body. Whether it has in addition other functions is at present in discussion. The biodiversity of the colonic microbiome is very high, particularly under healthy conditions, and includes multiple pathogens [1]. A protective mucosal barrier constrains bacteria and their toxic bacterial metabolites to the lumen. Although the concentrations and diversity of microbiota in small intestine is lower than in the large intestine, it may still suffice to express metabolic activity with potential impact for the host. Moreover, the compositions of the small and large intestinal microbiota are different, which may also impact on metabolism [1]. This is due to the proximity of the various bacterial species and their interaction in consolidated stool, the impact of the different metabolic milieu, the presence of bile acids in the small intestine (but not in colon), and potential secretory factors of the host small intestine. The microbiome of the small intestine fulfils specific metabolic functions that are different from their effects observed in the large intestine where absorption is negligible. Therefore, we postulated that the small intestinal microbiota produces bacterial breakdown products from food constituents, which are absorbed there. They may serve normal physiological functions in controlling metabolism, or cause harm in case of pathological conditions. The latter consideration is of significance in decompensated liver cirrhosis, where the commensal small intestinal microbiota generates ammonium from amino acids, which after absorption cannot be cleared by the liver due to functional failure or portocaval shunts, thus inducing hepatic encephalopathy. This is likely a small intestinal event because of the physiology of absorption, as no non-absorbed amino acids are left over in the colonic lumen, available for ammonium production and consequent absorption. However, this simple assumption that absorption occurs only in the small intestine is often neglected and textbooks still propose the colon as an absorptive organ, also for ammonium.

Recently, it was reported that in the gut, the bacterial breakdown of choline-containing nutrients, mainly lecithin, generates trimethylamine (TMA) in the human liver [2, 3]. This is strictly dependent on the microbiota and not on the host metabolism. The intestinal bacterial species responsible for phosphatidylcholine (PC) breakdown are not completely defined. Some species of Bacteroidetes (particularly *B*. *thetaiotaomicron* and *B*. *fragilis*) may be involved in TMA formation because they express phospholipases that hydrolyse dietary PC to choline [4]. In addition, it was reported that bacteria from the Erysipelotrichia class (Firmicutes phylum) can produce TMA from choline [5]. TMA itself is absorbed and readily oxidized to trimethylamine-N-oxide (TMAO) by hepatic flavin-containing monooxygenases (FMOs) [6].

Thus, TMAO is an indirect measure of the generated PC breakdown product TMA in the gut. Increased plasma levels of TMAO were associated with an increased risk of adverse cardiovascular events [7, 8], which has serious health implications, but also presents the option of preventing vascular damage by consuming a diet low in lecithin. It was indeed shown that the administration of oral broad-spectrum antibiotics suppressed plasma TMAO levels [8]. However, the sites of action were postulated as being the cecum and large intestine. This contention is challenged by data presented in this report, where the generation and absorption site of the PC breakdown product TMA was evaluated after exposure of a healthy volunteer to native and delayed-release PC preparations. It indeed showed that TMA is produced and absorbed only by the small intestine.

## Materials and Methods

### PC application to proband

For this proof-of-principle study, an individual healthy volunteer (male, 60 years, 185 cm, 82 kg, no disease, no medication, no diet) was chosen to reduce individual differences in metabolism determined by the genetic make-up, dietary habits, and microbiota. He maintained a diet low in phosphatidylcholine over the entire study period. According to the dietary analytical protocol [9], the total daily intake of PC corresponded to a choline content of <100 mg daily. For breakfast, the volunteer ate 2 sandwiches with marmalade or honey, tomatoes, or sausage. For lunch, he consumed a vegetable dish. Dinner was not provided. All drinks except for milk were allowed.

The volunteer was provided with 30 g lecithin (Allcura GmbH, Wertheim, Germany)—containing 6.9 g PC—at day 1 on an empty stomach. Blood and urine (12-h collection periods) were taken at time 0 and every subsequent 12 h for up to 48 h. Samples were stored at −80°C before analysis. In preceding experiments conducted over 72 h, it was shown that baseline levels were reached at 48 h after PC exposure. Lecithin was provided either native (i.e., without any antibiotic therapy) or during concomitant treatment with rifaximin (Xifaxam^®^; Norgine, The Netherlands; 3 doses of 550 mg each) 3 days before, as well as during the study period. Xifaxam^®^ is an intestinal topical acting antibiotic [10].

For comparison purposes, a delayed-released PC formulation was prepared using an enteric coating [11]. Pellets of native lecithin were coated by standard procedures in a fluidized-bed coating apparatus [12]. The coating solution (aqueous dispersion of Eudragit^®^ S100; Röhm Pharma GmbH, Germany) was sprayed onto the pellets at a flow rate of 0.8 mL/min. Eudragit S100 is a methacrylic acid copolymer that is soluble at pH 7.0. The coated pellets were stable when incubated at pH 2.0 (tested for up to 90 min) and dissolved in phosphate-buffered saline (pH 7.0 at 37°C, starting at 10 min and ending after 180 min). A preparation with a total of 6.9 g single PC application was used for this study.

The participant provided written, informed consent. The study and consent procedure were approved by the ethics committee of the University of Heidelberg (Ref. #S-453/2013).

### Determination of TMAO and TMA levels in plasma

TMAO and TMA were analysed by electrospray ionization tandem mass spectrometry (ESI-MS/MS) as described previously, with some modifications [13, 14]. Briefly, 20 μl of stable isotope-labelled standard 100 μM d^9^-TMA (C/D/N Isotopes, Inc., Quebec, Canada), 100 μM d^9^-TMAO (Sigma Aldrich, Taufkirchen, Germany), and 110 μL methanol (Roth, ROTIPURAN > 99,9%, Karlsruhe, Germany) were added to 50 μl plasma. After a centrifugation step (11,336 gmax, 5 min), 50 μl of the supernatant was transferred to an Eppendorf tube, and 4 μl of concentrated ammonium solution and 60 μl of ethyl bromoacetate 20 mg/ml in acetonitrile (Sigma Aldrich) were added. After 30 min, 90 μl of acetonitrile/water/formic acid (50: 50: 0.025; v/v/v) was added to the samples, and 20 μl of this solution was injected into the ESI-MS/MS system.

ESI-MS/MS analysis was performed using a Quattro Ultima triple-quadrupole mass spectrometer (Micromass, Manchester, UK) equipped with an electrospray ion source and a Micromass MassLynx data system. The ion source temperature was 100°C, and the desolvation temperature was 250°C. Data was obtained by multiple reaction monitoring: m/z 75.9/58.0 for TMAO and m/z 84.6/66.0 for d9-TMAO; m/z 146.1/118.1 for TMA and m/z 155.1/127.1 for d9-TMA. The collision gas was argon, and the collision energy was 20 eV for TMAO and 31 eV for TMA. The run time per sample was 2 min, and the mobile phase was 50:50 (v/v) methanol/water. Concentrations were calculated based on the signals relative to the internal standard and a 6-point calibration curve (0–100 μmol/L).

### Determination of free and total TMA in urine

TMA was determined by Solid-Phase-Micro-Extraction (SPME) headspace analysis of heated, alkalinized urine using gas chromatography mass spectrometry (GC/MS), as described previously [14, 15]. Briefly, 200 μL of 200 μM stable isotope-labelled d9-TMA (C/D/N Isotopes Inc., Quebec, Canada) was added as an internal standard to 100 μl urine. After the addition of 1 ml aqueous potassium hydroxide solution (6 M), the mixture was incubated in closed-headspace vials for 5 min at 50°C. The SPME fibre (Carboxen^™^-Polydimethylsiloxan, 75 μm; Supelco, Bellefonte, USA) was exposed to the headspace vapour for 10 min.

For GC/MS analysis, an MSD 5975A quadrupole mass spectrometer (Agilent Technologies, USA) was used with electron impact ionization. GC separation was achieved on a capillary column (DB-5MS, 30 m × 0.25 mm; film thickness: 0.25 μm; Agilent J&W Scientific, USA) using helium as a carrier gas. Extracted compounds were desorbed from the SPME fibre for 1 min in the GC/MS injector at 250°C in split-less mode.

The GC parameters used were 55°C for 3 min, ramping at 40°C/min to 180°C, and then ramping at 10°C/min to 300°C. The interface temperature was set to 230°C. The fragment ions studied for quantification were m/z 58 for TMA and m/z 66 for d9-TMA). TMAO was measured as total TMA by the same technique in a 2-step procedure, after oxide reduction by titanium (III) chloride (TiCl_3_). In the first step, 5 μl of TiCl_3_ (15% in 10% HCl) was added to 100 μl urine and the mixture was incubated in a closed headspace vial for 40 min at 37°C. In the second step, total TMA was determined as the sum of free and oxidized TMA. N-oxidation was expressed as the ratio of free to total trimethylamine in %.

### Statistical analysis

Data are expressed as the mean ± S.E.M., unless otherwise noted. Time-dependent changes were evaluated by 2-tailed analysis of variance (ANOVA), with repeated measures. Differing factors included differences in lecithin loadings (3 levels: lecithin, lecithin with rifaximin pretreatment, and delayed-release lecithin) and time (5 levels: 0, 12, 24, 36, and 48 h post-lecithin loading). Pairwise and multiple-comparisons paired t-tests with the Benjamini–Hochberg (BH) adjustment were calculated. All statistical analysis was performed in R environment for statistical computing [16]. p < 0.05 was considered as statistically significant, and p < 0.001 was considered highly significant.

## Results

After the application of each of the PC preparations (dose of 6.9 g), plasma and urinary TMA concentrations were not detectable in the plasma or were low (<9 mmol/mol creatinine, N-oxidation capacity > 90), respectively, and not statistically different between the examined conditions, indicating that TMA was rapidly oxidized to TMAO by the liver with normal FMO3 enzymatic activity [6]. The determined TMAO and TMA levels are summarized in Table 1.

**Table 1:** TMAO and TMA in plasma and urine after PC loadings.

Time (h)	PC			PC with rifaximin			PC delayed release		
	TMAO	TMA	N-Oxidation	TMAO	TMA	N-Oxidation	TMAO	TMA	N-Oxidation
Plasma	[μM]		%	[μM]		%	[μM]		%
0	10 ± 2	n.d.	-	8 ± 1	n.d.	-	10 ± 2	n.d.	-
12	43 ± 8	n.d.	-	22 ± 8	n.d.	-	20 ± 3	n.d.	-
24	27 ± 2	n.d.	-	13 ± 1	n.d.	-	9 ± 1	n.d.	-
36	24 ± 5	n.d.	-	15 ± 2	n.d.	-	9 ±2	n.d.	-
48	13 ± 2	n.d.	-	7 ± 1	n.d.	-	6 ± 1	n.d.	-
Urine	mmol/mol Creatinine		%	mmol/mol Creatinine		%	mmol/mol Creatinine		%
0	91 ± 20	5 ± 1	95	60 ± 5	5 ± 1	92	70 ± 8	5 ± 0	94
12	161 ± 21	8 ± 2	95	133 ± 31	7 ± 1	95	143 ± 18	7 ± 1	95
24	252 ± 33	7 ± 1	97	185 ± 31	9 ± 1	95	97 ± 9	6 ± 1	94
36	214 ± 26	8 ± 2	96	115 ± 26	7 ±1	94	74 ± 11	6 ± 1	92
48	127 ± 24	9 ± 2	94	71 ± 8	8 ± 2	90	55 ± 3	5 ± 1	91

Data are presented as the mean ± S.E.M. of 4–6 independent loading experiments. n.d.: not detectable (under the limit of quantification).

N-oxidation is expressed as the ratio of free to total trimethylamine in %.

Statistical analyses of TMAO and TMA levels determined in both body fluids (plasma and urine) focused on 2 different aspects: (1) changes of these compounds as a shape comparison over time (chronological sequence of increase, maximum and decrease, evaluation by 2-tailed ANOVA with repeated measures, p-values given in the text) and (2) comparison of the observed levels at each time point, depending on the different PC loadings. Results of the paired t-tests with pooled standard deviations and BH adjustments are given in S1 Table. The results of both ANOVA and paired t-tests can be summarized and interpreted as follows:

Comparison of the measured plasma TMAO values over time by ANOVA showed that all time courses were similar (p = 0.23) irrespective of the TMAO concentration. This finding indicated that flooding, peak excretion, and the decline to baseline TMAO levels after different PC loadings followed similar kinetics. TMA was found to be under the limit of quantification (Table 1).

**Fig 1:**
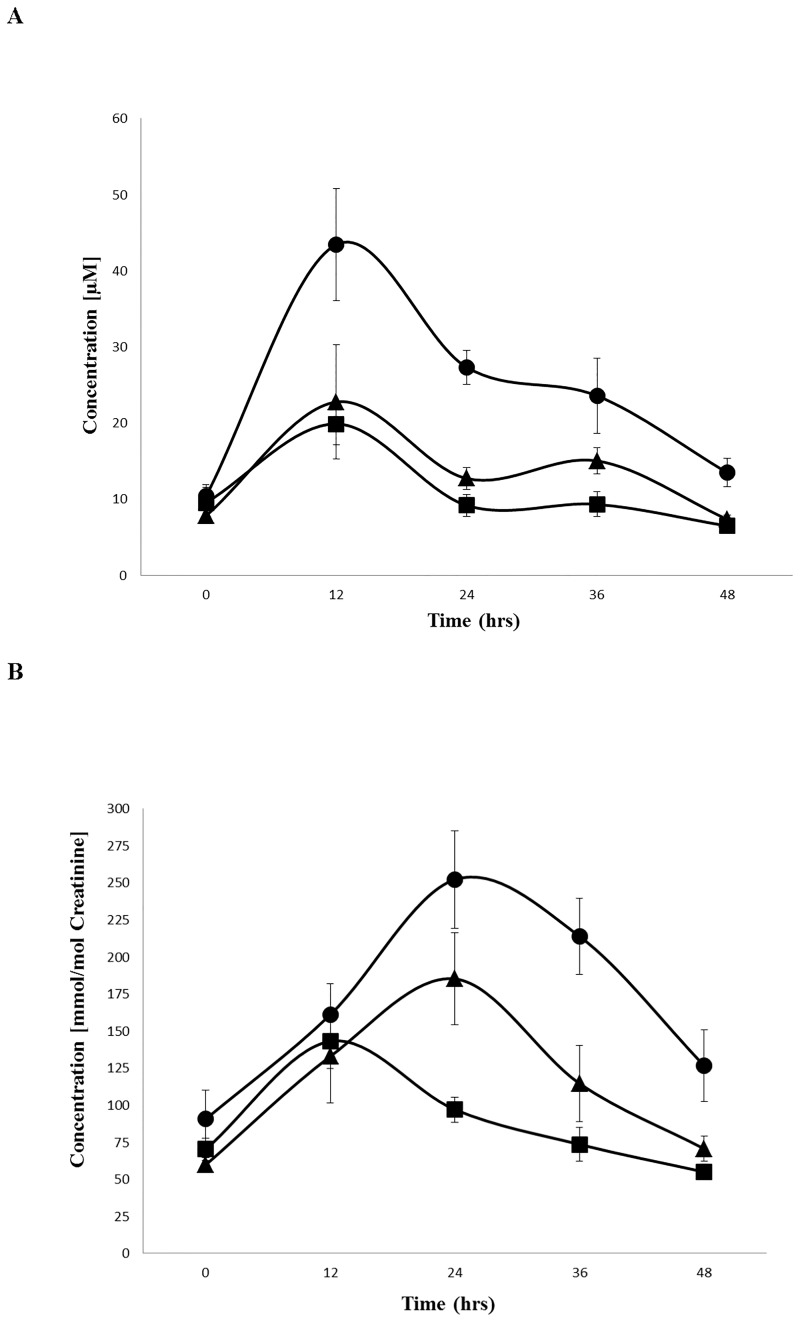
TMAO production in plasma (A) and urine (B)—a time course. Data are presented as the mean ± S.E.M. of 4–6 independent loading experiments. ● PC, ▲, PC with rifaximin pretreatment, ■ delayed-release PC

**Fig 2:**
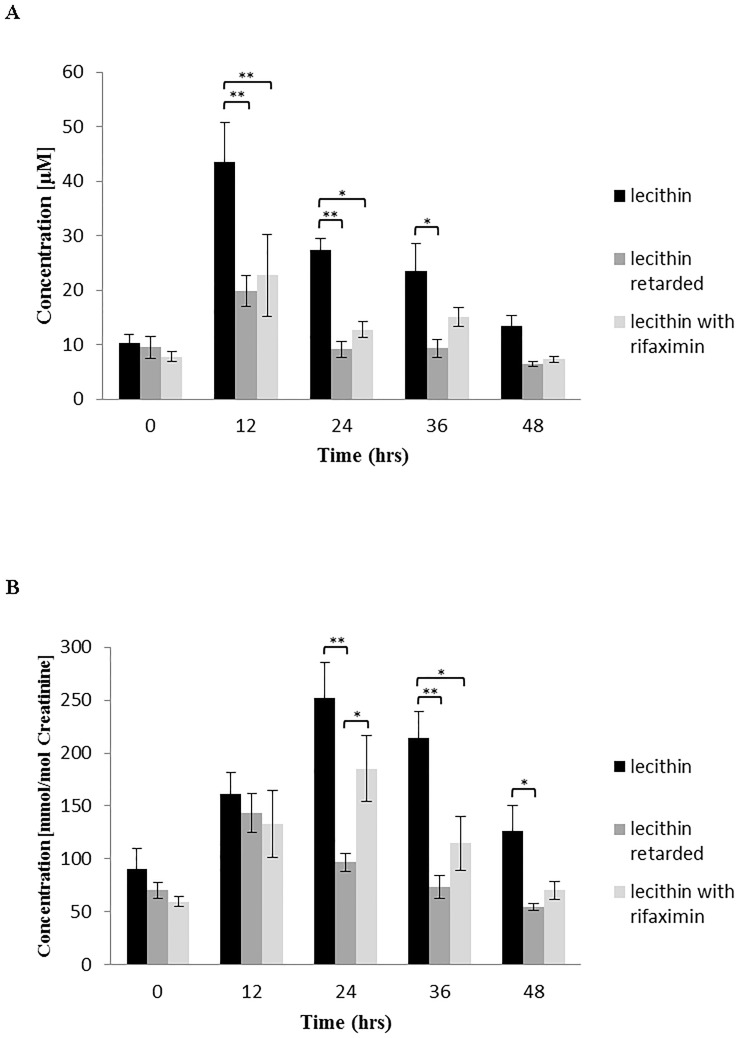
TMAO production in plasma (A) and urine (B)—statistical analysis. Data are presented as the mean ± S.E.M. of 4–6 independent loading experiments. *p < 0.05, **p < 0.001 (for details see S1 Table).

Under each loading condition (PC, PC with rifaximin pretreatment, and delayed-release PC), the TMAO plasma levels peaked 12 h after application (maximal level for native PC, 43 ± 8 μM) and dropped within 48 h to almost baseline values (Fig 1A). The concomitant treatment with rifaximin suppressed plasma TMAO levels significantly at all points in time (p < 0.001), with the most prominent relative inhibition observed at the 12-h time point (22 ± 8 μM, p < 0.001), as shown in Figs 1A and 2A. Delayed-release PC showed the lowest plasma TMAO levels, which were even significantly lower than PC in presence of rifaximin at 24 h (p < 0.001) and 36 h (p < 0.05) post-ingestion (Figs 1A and 2A).

The urinary TMAO levels followed the plasma disappearance kinetics with a delay of 12 h. After the initial 12-h urinary collection period, the TMAO concentration of all 3 PC preparations were identical at a low level. Thereafter, the TMAO concentrations were significantly (p < 0.001) suppressed by rifaximin pretreatment, and those were again reduced when delayed-release PC was provided (p < 0.001) at 24, 36 and 48 h after ingestion (Figs 1B and 2B).

## Discussion

In the present investigation, we addressed whether the absorption of bacterial breakdown products occurs in the small or large intestine, as well as the consequences for metabolism. Thus, we evaluated the generation of the PC-breakdown products TMA and its oxidized form TMAO after oral PC loading under different conditions.

Like any other basic food component (except fibres with ß-glycosylated bonds), PC is broken down by pancreatic enzymes to its monomeric components, i.e., by phospholipase A_2_ to lysosphosphatidylcholine (LPC) and a fatty acid, which are both absorbed in the upper small intestine. Therefore, the generation of bacterial breakdown products of PC occurs before the absorption process is completed in the upper small intestine, which implicates that bacteria are present at those sites. Indeed, suppression of the gut microbiota by the topical antibiotic rifaximin suppressed the bacterial production of PC-breakdown products significantly. In distal parts of the small intestine, PC is no longer present luminally. When delayed-release PC was provided, it bypassed the pancreatic enzymes and the full load of PC entered the distal small intestine and colon. There, the bacterial density is high, but the absorption of PC breakdown products is negligible, supporting the hypothesis that the colon is not capable of TMA absorption. Whether this applies also to other substrates needs to be evaluated, but appears to be likely. The recent development of delayed-release PC for the therapy of ulcerative colitis [11, 17] seems, in contrast to native PC, of no harm in regard to TMAO generation, as indicated by the results of this study.

The same mechanism of microbiota-dependent metabolic control applies to ammonium generation, which becomes of clinical relevance in patients with end-stage liver disease and/or portal-systemic shunts, who may develop hepatic encephalopathy. These patients also benefit from rifaximin therapy acting in the upper small intestine [10].

Whether elevated TMAO levels indeed indicate a risk for cardiovascular disease has to be evaluated in prospective, controlled interventional studies, which might be difficult to perform. Previously, PC was thought to be beneficial for human health. This has been challenged in recent reports [7, 8]. Whether patients at risk should take preventive measures to avoid a high TMAO load is another interesting question. Potential therapeutic options include the application of suitable antibiotics, or the enrichment of the constitutive intestinal bacterial flora with probiotic microbiota showing low PC-degrading characteristics (e.g. low phospholipase activity).

The clear cut results of this observational study are limited by a lack of direct data regarding PC metabolites in the gut, as well as detailed analysis of the microbiota. However, these consideration may not be essential for investigating the question addressed here. The fact that just 1 individual person was tested may also hamper the conclusions. Thus, the results of this study should stimulate further trials to prove this new and challenging view on absorption and the impact of the gut microbiota on metabolism.

## Supporting Information

S1 Table Statistical analysis of TMAO production in plasma and urine. (DOCX)
