# Supplementary material for: Blood Trimethylamine-N-Oxide Originates from Microbiota Mediated Breakdown of Phosphatidylcholine and Absorption from Small Intestine
Source: PLoS One. 2017 Jan 27;12(1):e0170742. doi: 10.1371/journal.pone.0170742 (PMC5271338; doi:10.1371/journal.pone.0170742)
Supplement: S1 Table — (DOCX) [file pone.0170742.s001.docx]

**Supplementary Table 1:** *Statistical analysis of TMAO production in plasma and urine.*

| Plasma | PCAB:0 | PCAB:12 | PCAB:24 | PCAB:36 | PCAB:48 | PC:0 | PC:12 | PC:24 | PC:36 | PC:48 | PCdr:0 | PCdr:12 | PCdr:24 | PCdr:36 |
| --- | --- | --- | --- | --- | --- | --- | --- | --- | --- | --- | --- | --- | --- | --- |
| PCAB:12 | 0.00537 |  |  |  |  |  |  |  |  |  |  |  |  |  |
| PCAB:24 | 0.43865 | 0.07713 |  |  |  |  |  |  |  |  |  |  |  |  |
| PCAB:36 | 0.20666 | 0.20103 | 0.74851 |  |  |  |  |  |  |  |  |  |  |  |
| PCAB:48 | 0.94580 | 0.00594 | 0.41059 | 0.20241 |  |  |  |  |  |  |  |  |  |  |
| PC:0 | 0.70104 | 0.02570 | 0.74851 | 0.48904 | 0.65882 |  |  |  |  |  |  |  |  |  |
| PC:12 | 1.7e-09 | 0.00030 | 1.1e-07 | 7.1e-07 | 2.9e-09 | 1.8e-08 |  |  |  |  |  |  |  |  |
| PC:24 | 0.00033 | 0.49700 | 0.00895 | 0.02655 | 0.00043 | 0.00258 | 0.00414 |  |  |  |  |  |  |  |
| PC:36 | 0.00352 | 0.90499 | 0.05515 | 0.14830 | 0.00398 | 0.01714 | 0.00044 | 0.58819 |  |  |  |  |  |  |
| PC:48 | 0.32699 | 0.08654 | 0.90499 | 0.82478 | 0.30954 | 0.65273 | 7.1e-08 | 0.00930 | 0.06108 |  |  |  |  |  |
| PCdr:0 | 0.80828 | 0.01714 | 0.65273 | 0.39720 | 0.76533 | 0.90499 | 1.0e-08 | 0.00158 | 0.01150 | 0.55459 |  |  |  |  |
| PCdr:12 | 0.01714 | 0.65882 | 0.20995 | 0.44387 | 0.01791 | 0.08147 | 1.5e-05 | 0.19923 | 0.58277 | 0.24661 | 0.05548 |  |  |  |
| PCdr:24 | 0.83334 | 0.01070 | 0.59810 | 0.33036 | 0.79489 | 0.86648 | 2.9e-09 | 0.00081 | 0.00753 | 0.48904 | 0.95772 | 0.03662 |  |  |
| PCdr:36 | 0.83231 | 0.01574 | 0.63839 | 0.37330 | 0.79489 | 0.88843 | 9.9e-09 | 0.00150 | 0.01066 | 0.52935 | 0.97174 | 0.05142 | 0.97862 |  |
| PCdr:48 | 0.83437 | 0.00258 | 0.29689 | 0.12490 | 0.90499 | 0.55459 | 9.7e-10 | 0.00013 | 0.00158 | 0.20284 | 0.65273 | 0.00895 | 0.65882 | 0.65882 |
| Urine | PCAB:0 | PCAB:12 | PCAB:24 | PCAB:36 | PCAB:48 | PC:0 | PC:12 | PC:24 | PC:36 | PC:48 | PCdr:0 | PCdr:12 | PCdr:24 | PCdr:36 |
| PCAB:12 | 0.03154 |  |  |  |  |  |  |  |  |  |  |  |  |  |
| PCAB:24 | 0.00043 | 0.12083 |  |  |  |  |  |  |  |  |  |  |  |  |
| PCAB:36 | 0.12083 | 0.61916 | 0.04646 |  |  |  |  |  |  |  |  |  |  |  |
| PCAB:48 | 0.76507 | 0.06540 | 0.00117 | 0.22114 |  |  |  |  |  |  |  |  |  |  |
| PC:0 | 0.41358 | 0.22114 | 0.00789 | 0.52429 | 0.60329 |  |  |  |  |  |  |  |  |  |
| PC:12 | 0.00421 | 0.42935 | 0.52429 | 0.20367 | 0.01007 | 0.04646 |  |  |  |  |  |  |  |  |
| PC:24 | 2.9e-06 | 0.00103 | 0.07475 | 0.00032 | 3.8e-06 | 2.8e-05 | 0.01452 |  |  |  |  |  |  |  |
| PC:36 | 1.3e-05 | 0.01184 | 0.42733 | 0.00346 | 3.2e-05 | 0.00034 | 0.12083 | 0.30679 |  |  |  |  |  |  |
| PC:48 | 0.06018 | 0.85496 | 0.10153 | 0.75125 | 0.11914 | 0.32768 | 0.34977 | 0.00093 | 0.01007 |  |  |  |  |  |
| PCdr:0 | 0.76507 | 0.06540 | 0.00117 | 0.22114 | 0.99247 | 0.60304 | 0.01007 | 3.8e-06 | 3.2e-05 | 0.11889 |  |  |  |  |
| PCdr:12 | 0.01328 | 0.76507 | 0.22114 | 0.42733 | 0.03160 | 0.12083 | 0.62108 | 0.00253 | 0.02999 | 0.64013 | 0.03154 |  |  |  |
| PCdr:24 | 0.28816 | 0.28438 | 0.00975 | 0.62446 | 0.45855 | 0.85496 | 0.06018 | 2.8e-05 | 0.00034 | 0.41358 | 0.45766 | 0.15830 |  |  |
| PCdr:36 | 0.69211 | 0.06540 | 0.00103 | 0.23365 | 0.92521 | 0.63279 | 0.01007 | 3.8e-06 | 2.8e-05 | 0.12083 | 0.92521 | 0.03154 | 0.49681 |  |
| PCdr:48 | 0.89235 | 0.01472 | 0.00016 | 0.07822 | 0.65777 | 0.30889 | 0.00171 | 1.2e-06 | 3.8e-06 | 0.03356 | 0.65832 | 0.00671 | 0.20644 | 0.60304 |

Pairwise comparisons using paired t test with pooled standard deviation and Benjamini-Hochberg adjustment. PCAB: PC with refaximin; PCdr: PC delayed release; PC: PC; PCAB 0 – 48 h: PC with refaximin at time point 0 – 48 h; PCdr 0 – 48: PC delayed release at time point 0 – 48 h; PC 0 – 48: PC at time point 0 – 48 h.
